# From tumors to species: a SCANDAL hypothesis

**DOI:** 10.1186/s13062-019-0233-1

**Published:** 2019-01-23

**Authors:** A. Y. Panchin, V. V. Aleoshin, Y. V. Panchin

**Affiliations:** 1 Institute for Information Transmission Problems, Bolshoy Karetniy Pereulok 19/1, Moscow, Russian Federation 127051; 2 A.N. Belozersky Institute of Physico-Chemical Biology, Moscow State University, Moscow, Russia

**Keywords:** Evolution, Cancer, Transmissible tumor, Speciation, Myxosporea, Myxozoa, Simplification, Devil facial tumor disease

## Abstract

**ᅟ:**

Some tumor cells can evolve into transmissible parasites. Notable examples include the Tasmanian devil facial tumor disease, the canine transmissible venereal tumor and transmissible cancers of mollusks. We present a hypothesis that such transmissible tumors existed in the past and that some modern animal taxa are descendants of these tumors. We expect potential candidates for SCANDALs (**s**peciated by **can**cer **d**evelopment **a**nima**ls)** to be simplified relatives of more complex metazoans and have genomic alterations typical for cancer progression (such as deletions of universal apoptosis genes). We considered several taxa of simplified animals for our hypothesis: dicyemida, orthonectida, myxosporea and trichoplax. Based on genomic analysis we conclude that Myxosporea appear to be the most suitable candidates for a tumor ancestry. They are simplified parasitic cnidarians that universally lack major genes implicated in cancer progression including all genes with Caspase and BCL2 domains as well as any p53 and apoptotic protease activating factor – 1 (Apaf-1) homologs, suggesting the disruption of main apoptotic pathways in their early evolutionary history. Further comparative genomics and single-cell transcriptomic studies may be helpful to test our hypothesis of speciation via a cancerous stage.

**Reviewers:**

This article was reviewed by Eugene Koonin, Mikhail Gelfand and Gregory M Woods.

**Electronic supplementary material:**

The online version of this article (10.1186/s13062-019-0233-1) contains supplementary material, which is available to authorized users.

## Background

Tumors result from an evolutionary process with selection acting at the level of somatic cells within the organism [1, 2]. In some scenarios, this evolution proceeds beyond the original tissue through metastases and even beyond the original body in the form of transmissible cancers. The most famous example is the Tasmanian devil facial tumor disease [3] presented by two clones [4]. Other examples include the canine transmissible venereal tumor (CTVT) [5] and the contagious reticulum cell sarcoma of the Syrian hamster [6] (although it is possible that the tumor had a viral origin [7]). Four independent transmissible cancers were found in bivalve mollusks (*Mya arenaria*, *Mytilus trossulus*, *Cerastoderma edule* and *Polititapes aureus*) [8, 9]. Notably, in the case of *P. aureus* the cancer was attributed to a non-host species [9]. This suggests that transmissible cancers are not uncommon in nature.

Several recent papers considered transmissible cancers in the light of general evolution and oncology and identified multiple genetic adaptations for their long-term survival and efficient transfer [5, 7]. Given that at least one tumor, CTVT, has existed for thousands of years [5, 10] and outlived its progenitors, it is possible for tumor cells to evolve into a new species.

Our hypothesis is that some simplified relatives of complex metazoans can have a tumor origin. Abrupt simplification via cancerous transformation and speciation through the acquisition of transmissibility predicts massive loss of systems, functions, cellular processes and related genes at the initial cancer stage followed by gradual evolution and adaptation to a parasitic lifestyle. Potential candidates for SCANDALs (**s**peciated by **can**cer **d**evelopment **a**nima**ls)** are expected to be simplified relatives of more complex metazoans, acquire multicellularity de novo and to lack crucial genes that are involved in apoptosis and tumor suppression.

Modern cancer genetics studies [11], including single cancer cell sequencing [12] have identified genes and pathways that are typically disrupted during cancer progression. Hallmarks of cancer neoplasia include proliferation of genetically altered cells that fail to respond to normal regulatory controls of cell growth, self sufficiency in growth signals, insensitivity to antigrowth signals, evasion of apoptosis, unlimited potential to replicate, tissue invasion and metastasis [13]. The most important tumor-suppressing genes are expected to be lost during the initial stages of catastrophic simplification and as a result they should be absent in all representative species of a potential SCANDAL group.

We considered four simplified (non-basal) multicellular groups of Metazoa with available complete genomes or high throughput sequencing data for the role of SCANDALs: Dicyemida, Orthonectida, Myxosporea and Trichoplax. Phylogenetic analysis shows that all four groups derived from ancestors that were more complex [14].

*Trichoplax* spp. is a simple, small, flattened, free-living animal from the phylum Placozoa [15]. Dicyemida (also known as Rhombozoa) are a phylum of simplified parasitic animals that live in the renal appendages of cephalopods [16]. Myxosporea are a group of aquatic obligatory parasitic cnidarians [17–19]. Their morphology is very different from normal Metazoa. Among the stages of their life cycle there is a three-cell four-nucleus stage, a single-cell single-nucleus stage, a multicellular plasmodium stage and even a multicellular plasmodium with cells in cells stage. Myxosporea infest two types of hosts: fish and invertebrates (usually annelids) [20]. Orthonectida were historically considered very simple parasitic organisms, however recent works have shown that they have nervous and muscular systems [21], which makes them the least probable candidates for a tumor origin. We investigated whether the genetic alterations in these groups are consistent with our hypothesis.

## Methods

Using HMMER (http://hmmer.org/) we identified PFAM domains (https://pfam.xfam.org/) of 9 potential SCANDALs – 5 Myxosporea (*Thelohanellus kitauei, Kudoa iwatai, Myxobolus cerebralis, Sphaeromyxa zaharoni, Enteromyxum leei*), Placozoa – *Trichoplax adhaerens,* Dicyemida *– Dicyema* sp., Orthonectida *– Intoshia linei.* 29 other Metazoa species *Hydra vulgaris, Nematostella vectensis, Acropora digitifera, Polypodium hydriforme, Echinococcus granulosus, Gyrodactylus salaries, Schistosoma mansoni, Adineta vaga, Drosophila melanogaster, Eurytemora affinis, Strigamia maritime, Ixodes scapularis, Peripatopsis capensis, Gordionus alpestris, Hypsibius dujardini, Ascaris suum, Toxocara canis, Caenorhabditis elegans, Romanomermis culicivorax, Priapulus caudatus, Saccoglossus kowalevskii, Strongylocentrotus purpuratus, Ciona intestinalis, Homo sapiens, Branchiostoma floridae, Mnemiopsis leidyi, Pleurobrachia bachei, Amphimedon queenslandica, Oscarella carmela*, and 3 unicellular Holozoa species *– Monosiga brevicollis, Capsaspora owczarzaki* and *Sphaeroforma arctica*.

Published proteome data was used when available, but for some potential SCANDALs (*Dicyema* sp., *Kudoa iwatai, Myxobolus cerebralis, Sphaeromyxa zaharoni, Enteromyxum leei*) only nucleotide data (genomic or transcriptomic) was found in NCBI databases. In these cases we performed a search for all coding ORFs in six frames (length > 20 amino acids). Eventually we generated and analyzed a database of PFAM domains for 41 species. Then we investigated the presence of PFAM domains associated with genes that have been previously described as hallmarks of cancer neoplasia [22] in candidate and control species. For this we used the PFAM list from a “census of human cancer genes” [22] and also performed a PFAM search for the term “apoptosis”. We used 409 domains that were present in either of the two lists and present in 15 or more of 29 the control Metazoan species.

## Results

### Initial hypothesis testing

**Fig. 1:**
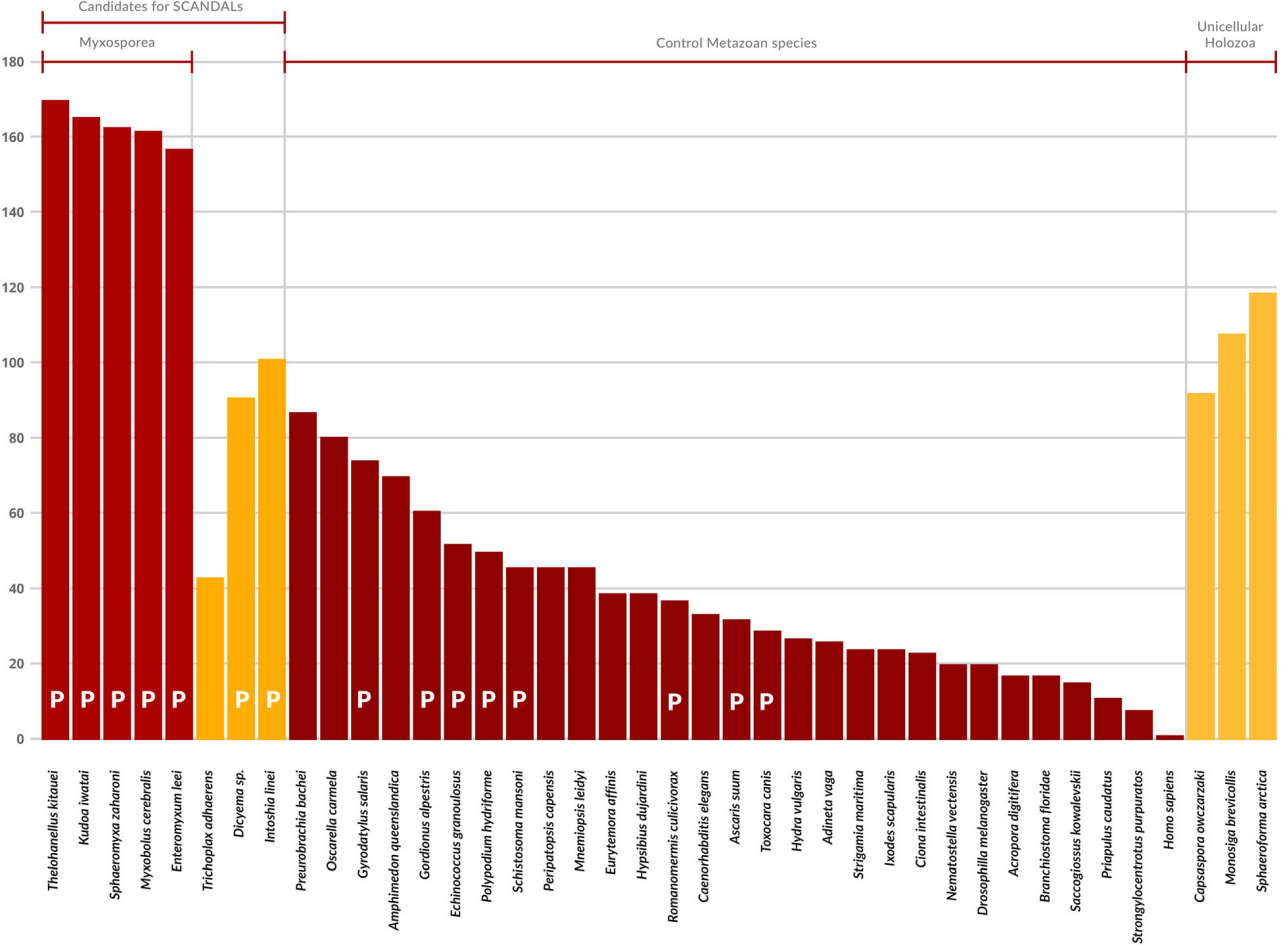
A comparison of SCANDAL candidates and control species in terms of PFAM domains. We used the PFAM list from a “census of human cancer genes” [22] and performed a PFAM search for the term “apoptosis”. We used 409 domains that were present in either of the two lists and present in 15 or more of the 29 control Metazoan species. The number of such domains absent in each species is shown on the y-axis. Parasitic species are marked by P

The five Myxosporea species turned out to have lost the largest number of PFAM domains associated with apoptosis or cancer comparing to other SCANDAL candidates and control species, including the simplified parasitic cnidarians *Polypodium hydriforme* (closely related to Myxosporea) and unicellular basal Holozoans (Fig. 1).

One of the main hallmarks of cancer progression is the disruption of apoptotic pathways. Proteins with Caspase and BCL2 domains are essential for apoptosis [23]. These domains are universal for Metazoa and the Caspase domain is even found in basal unicellular Holozoa (Additional file 1: Table S1). Therefore, we predicted that genes encoding these domains would be lost in SCANDALs. Genomic analysis revealed that this prediction does not hold for Dicyemida, Orthonectida and Placozoa. However, we were unable to find any BCL2 or Caspase domains encoded in any of the Myxosporea genomes, transcriptomes or proteomes. This indicates that these domains were lost early during their evolution.

As our initial prediction failed with Dicyemida, Orthonectida and Placozoa, we did not further investigate their tumor origin. We investigated the presence of other cancer-related genes and PFAM domains in Myxosporea in detail.

### Genomic analysis of Myxosporea

Homologues of Caspases, BCL2 and Apaf-1 are core proteins necessary for apoptosis and are universally present in both vertebrates and invertebrates. PFAM domains characteristic for these proteins Peptidase_C14 (PF00656) Caspase, Apoptosis regulator proteins domain Bcl-2 family (PF00452) and Caspase recruitment CARD domain (PF00619) present in Apoptotic protease activating factor – 1 (Apaf-1) proteins are absent in all five studied species of Myxosporea. These crucial PFAM domains are encoded by the genomes of all other studied cnidarians including *Polypodium hydriforme* that is also a parasitic animal and is the closest phylogenetic relative of Myxozoa [17–19, 24].

Calpains are a group of proteases that are activated by increased intracellular calcium levels. They have been implicated in apoptotic cell death, and necrosis [25]. The Calpain family cysteine protease (PF00648) with Calpain large subunit domain III (PF01067) is universally present in metazoans and is even found in some unicellular Holozoans such as *Monosiga brevicollis* and *Capsaspora owczarzaki*, but are not detected in any Myxosporea.

The Death domain (PF00531) is present in proteins that interact with caspases and NF-kappaB, that are involved in the regulation of apoptosis and inflammation. Death domains are not found in Myxosporea and Holozoa species, but they are almost ubiquitously present in all other Metazoa (we have not detected the Death domain only in *Gyrodactylus salaries* in our dataset). PF06905 Fas apoptotic inhibitory molecule (FAIM1) is also found in most Metazoa species but not in Myxosporea. PF00554 NF-kappaB-RHD_DNA_bind domain is absent in Myxosporea.

Misregulation of Wnt signaling can lead to tumor development via excessive cell proliferation. Wnt pathways are activated by binding of the Wnt-protein ligand to a Frizzled family receptor, which passes the biological signal to the Dishevelled protein inside the cell. The absence of the Wnt pathway key components was previously reported for two members of Myxosporea: *K. iwatai* and *M. cerebralis* [24]. Although some researchers claim that *T. kitauei* carries genes encoding components of Wnt pathways [26] we discovered the loss of PFAM domains associated with the Wnt pathway in *T. kitauei* and four other Myxosporea. Wnt ligands (PF00110 Wnt family) are ubiquitously present in all Metazoa (but not in unicellular Holozoa) and are lost in all five studied Myxosporea. WIF domain (PF02019), Frizzled/Smoothened family membrane region (PF01534), and Dishevelled (PF02377) are lost in all five studied Myxosporea.

All Myxosporea lack the PF00870 p53 DNA-binding domain. This domain is found in many animals including free-living Сnidaria and the parasitic *Polypodium hydriforme*, and even in some unicellular Holozoans such as *Monosiga brevicollis*, *Capsaspora owczarzaki* and *Sphaeroforma arctica*. However, quite a few metazoans including Orthonectida also lack this domain. p53 is a tumor suppressor in humans and many other species [27]. p53 is activated by DNA damage and turns on several downstream pathways that may induce cell cycle arrest, apoptosis and DNA repair.

Among other PFAM domains lost in Myxosporea but universally present in Metazoa is PF03134, family TB2/DP1 (deleted in polyposis). This family contains the HVA22 protein which is deleted in severe forms of familial adenomatous polyposis. This is an autosomal dominant oncological inherited disease in humans [28].

### Myxosporea: A detailed hypothetical scenario of speciation through cancerogenesis

Myxosporea belong to a class called Myxozoa that contains another sub-class – Malacosporea. The latter group is morphologically more complex [29]. For example, *Buddenbrockia* (Malacosporea) have a vermiform life cycle stage [30], appear to have myocyte cells [31] and reportedly have a life cycle stage similar to a blastula that undergoes a process similar to gastrulation [32]. No signs of embryonic development, blastula or gastrulation have been identified in Myxosporae so far.

Thus, the SCANDAL hypothesis is more likely to be true for Myxosporae than for Malacosporea. Genomic studies of Malacosporea could help distinguish between catastrophic (abrupt) oncogenic simplification and gradual simplification. Unfortunately, Malacosporea genomes have not been sequenced.

Recent molecular phylogenomic studies [24, 33] place Myxosporea among Cnidaria. Although we found no reports of transmissible tumors in Cnidaria, some cases of regular tumorigenesis were reported for this animal group [34]. Calicoblastic neoplasms have been found in corals and are characterized by rapid growth, loss of differentiation, loss of tissue architecture, proliferation of gastro-vascular canals and fitness reduction [35, 36]. Tumors were also recently identified in *Hydra* [37]. A transriptome analysis suggested that some of the misregulated genes of these tumors are homologous to mammalian tumor-related genes, including those involved in apoptosis [37]. The cells in these tumors were invasive and could be viewed as metastatic.

Inside Cnidaria Myxozoa are considered to be most closely related to *Polypodium* [17–19, 24]. *Polypodium* is also a fish parasite, but reproduces through complex free-living medusoid-like forms. Also, unlike *Polypodium*, fish-to-fish transmission of Myxosporeans by oral ingestion was reported [38]. It is possible that ancestral Myxosporeans used this type of dissemination. Transmissible tumor cells and especially those capable of interspecies transmission need to develop necessary defensive mechanisms to avoid the host’s immune system. Since Myxosporea are parasites of fish and their close relatives such as *Polypodium* are also parasites of fish, such adaptations could have been preexisting in their last common ancestor.

Many biological manifestations of cancers and parasitic infestations are similar. Cachectic syndrome is a common attribute of cancers and at the same time a feature of fish pathology caused by Myxosporea [39]. However, it is not typical for parasites (but typical for cancers) to lose genes that control cell grows and proliferation as in the case of Myxosporea.

Cnidaria-specific stinging organelles – cnidocysts or cnida are present in the cells of Myxosporea species [20]. This may hint on the initial type of cells from which the putative cancer originated. Cancer cell lines tend to retain features of their original precursor. For instance the HeLa cell line (considered as a novel species by some researchers [40]) retained similarity to ancestral uterus cervical cells on the transcriptomic level [41] while the Tasmanian devil transcriptome reveals Schwann cell origins of the transmissible cancer [42]. In the case of Myxosporea we could hypothesize that they originated from totipotent migratory stem cells called i-cells [43] or more specialized precursors of ectoderm cnidoblast cells or cnidoblast cells that restored the ability to proliferate. The proteomics of the cnidoblast are now described [44, 45] and we know that Myxosporea express nematocyst structural minicollagens and nematogalectins [33, 46, 47] that are encoded by taxonomically and tissue restricted genes. Perhaps, single-cell transcriptomics of different Cnidaria cell types will resolve the question, whether Myxosporea have evolved from this cell type or disprove our hypothesis.

Thus, we can put forward several hypothetical steps of Myxosporea evolution. First, a somatic cell in a parasitic *polypodium*-like organism lost some genes related to apoptosis and cell-cycle control and became cancerous. The tumor spread to a fish or annelid host (similar to a recent case how a *Hymenolepis nana* flatworm cancer spread to a human host [48]). This tumor acquired transmissibility as in known mammalian and mollusk transmissible cancers. Two different evolutionary trends followed and produced the modern Myxosporea. One trend was caused by the cancerous origin of Myxosporea species: multiple genes involved in apoptosis, cell-cycle suppression and associated pathways were abruptly lost. A different trend was directed towards a de novo formation of multicellularity resulting in the currently observed strange organisms with three-cell stages and bizarre cell aggregates like syncitia with whole cells inside other cells. It was suggested, that multicellularity evolved independently at least 25 times in eukaryotes [49, 50], which indicates that such scenarios are not particularly rare. These hypothetical events of de novo multicellularity and adaptation to parasitism probably resulted in additional peculiarities of this group. For instance aquaporins (PF00230 Major intrinsic protein) that are universal not only to metazoans but also to all cellular organisms are absent in Myxosporea.

### Limitations of the SCANDAL hypothesis for Myxosporea

The strongest support for our cancer speciation hypothesis comes from the absence of some universal PFAM domains in Myxosporea. Based on this evidence, we predict that Myxosporea should lack apoptosis. However, it is possible that some domains were lost due to insufficient sequencing coverage and assembly errors. To minimize these risks we used all available Myxozoa high throughput sequencing data from five species and additionally analyzed the PFAM domains that were lost in all five analyzed species by BLAST. In several cases, apparent fish host contaminations were removed. The studied domains are usually present in multiple copies, which also reduces the risk of false negative results.

The ancestors of Myxosporea experienced a degeneration of their body plan from that of a free-living Cnidaria to a parasite. This was accompanied by an overall reduction of genome size and gene content [24]. While this would be expected under the SCANDAL hypothesis, we cannot exclude the possibility that the observed loss of genes related to apoptosis and cancer is just a byproduct of overall Myxosporea simplification. We must admit as evidence against our hypothesis, that while some important cancer signaling pathways are absent in Myxosporea, not all of them are lost and we did not find that cancer-related domains were lost at a higher rate, than other domains. However, we do not observe genomic simplification of such scale in related parasites such as *Polypodium* and Metazoan parasites in general.

One even more exotic proposal on Myxosporea evolution could be derived from Shostac’s symbiogenetic hypothesis on Cnidaria origin [51]. The author speculated that cnidocysts, or cnida, that are a hallmark cell organelle of Cnidaria were acquired during their early evolutionary history from extrusive organelle-bearing protists living as symbiotic partners with some ancient Metazoan. In this case Myxosporea could be viewed as such extrusive organelle-bearing protists or as a result of fusion between two species (similar to mitochondrion acquisition [52]) that developed multicellularity. Current phylogenomic studies show no support for this view and confirm that Myxosporea are not basal Cnidaria and most likely derived by simplification from more complex animals (gradually or catastrophically). It now appears that the ballistic nematocysts in Cnidaria (including Myxosporea) evolved independently from extant extrusive organelles-bearing protoctistans [53].

Even if our SCANDAL hypothesis about Myxosporea evolution is incorrect, it may still apply to some other simplified metazoans. Future sequencing of additional potential SCANDALs and possible discovery of new peculiar Metazoan species may provide new insights into this matter.

## Conclusions

Among four putative groups of SCANDAL candidates Myxosporea appear to be the most promising. Their evolution involved the elimination of multiple genes related to apoptosis and cancer suppression, including up- and down- stream players of corresponding pathways. Further comparative genomics and single-cell transcriptomic studies may help test our hypothesis of speciation via a cancerous stage. Meanwhile it remains an interesting possibility that warrants additional investigation.

## Reviewers comments

### Reviewer 1: Dr. Eugene Koonin

Panchin et al. propose the SCANDAL hypothesis under which certain groups of secondarily simplified animals, in particular, Myxosporea, evolved from transmissible cancers. The hypothesis is both obvious enough - after all, the transmissible tumors are a step ahead of metastases on the path of tumor autonomization, and it is natural to speculate on the next step - and scandalous enough which, I suppose, is a good feature for a hypothesis. The discussion in the article is well informed, with respect to both transmissible tumors and animal evolution, and interesting. The authors make a laudable attempt to render the hypothesis falsifiable by postulating that any serious candidates for the origin from trnasmissible tumors should have lost a substantial fraction of tumor suppressors and apoptosis effectors. This is a (overly?) stringent criterion because there are many pathways to cancer, not necessarily through currently known “cancer genes”, and many ways to impair apoptosis as well. So, in a sense, this amounts to searching under the streetlight, but I think the approach is appropriate as a strong selection criterion for dismissing SCANDAL candidate. Indeed, by applying it, the authors discard 3 of the 4 initially suggested groups, and zero in on Myxosporea that have indeed lost quite a few of the genes of interest. As the authors admit, this cannot be really considered “support” for the hypothesis, only observations that appear compatible with it. Again, as the authors rightly note, the loss of these genes seems to be part of the general trend of reductive evolution, with no evidence that it was selective with respect to the cancer-related genes. My main question is very simple: do Cnidaria have cancer? If so, even if transmissibility has not been demonstrated, then, the origin of Myxosporea from a tumor would appear a distinct possibility. If not, the entire scenario is entirely speculative. On the whole, I tend to think that the hypothesis is wrong, and there is no SCANDAL in the animal kingdom. However, the possibility that there is definitely merits discussion, so the article will be of interest to many and could stimulate new insight and actual new research.

#### Authors response

We thank Dr. Koonin for his comment on our hypothesis. We decided that the answer to the question “do Cnidaria have cancer?” should be better presented in the main text of our article so we added the following paragraph:

**“**Calicoblastic neoplasms have been found in corals and are characterized by rapid growth, loss of differentiation, loss of tissue architecture, proliferation of gastro-vascular canals and fitness reduction [35, 36]. Tumors were also recently identified in *Hydra* [37]. A transriptome analysis suggested that some of the misregulated genes of these tumors are homologous to mammalian tumor-related genes, including those involved in apoptosis [37] The cells in these tumors were invasive and could be viewed as metastatic**”.**

### Reviewer 2: Dr. Mikhail Gelfand

The hypothesis presented in the paper is likely wrong, but clever and interesting. I commend the authors for citing not only supporting evidence (loss of many cancer-relate domains), but evidence against it (retention of some domains and same rates of loss in cancer-related and control domains).

The only technical point I can make relates to identification of domains via analysis of ORFs (in the absence of published annotation). Could it be that the exon length in some species is so small that domains might have been missed by the applied procedure? A control could be systematic search for some other domains using the same procedure. Similarly, to be on the safe side, it might be a good idea to check for possible underannotation by searching for the missing domains in Myxosporea genomes directly, not relying on annotation. The authors mention hamster reticulum cell sarcoma as an example of transmissible cancer. This cancer is not mentioned in a number of recent reviews (e.g Ujvari, Papenfuss & Belov, Bioessays, 2016 and Riquet, Simon & Bierne, Evol. Appl., 2016). The most likely explanation is that the authors of these reviews have missed old publications, in particular, the 1964 one cited by the authors. However, there seems to be some conflicting evidence: while a 1965 paper (Banfield, Woke, Mackay & Cooper, Science, 1965) states that mosquitoes transmit cancer cells and not any other oncogenic agent, a 1973 paper suggests that reticulum cell sarcoma of Syrian hamsters is caused by SV40 virus (Diamandopoulos, J. Natl. Cancer Inst., 1973). Could that be a different type of this cancer, or could the authors of the 60’s papers miss this possibility? In interesting issue, not directly covered by the authors, is what genes should have evolved (or re-evolved) to regain multicellularity of Myxosporea, if the hypothesis of their cancer, unicellular origin is true.

The sentence “multicellularity evolved independently at least 46 times in eukaryotes” clearly contains a misprint. The grammar needs to be checked, e.g. some commas and articles are clearly redundant.

#### Authors response

We thank Dr. Gelfand for his suggestions and questions.

The five Myxozoa species used in our analysis had different types of high-throughput sequencing data available. For three species (*Kudoa iwatai*, *Sphaeromyxa zaharoni* and *Enteromyxum leei*) we had genomic data, for one (*Myxobolus cerebralis*) we used transcriptome data and proteome data was available for the final species (*Thelohanellus kitauei*). We obtained similar results of domain loss for all five species. Thus, it is unlikely that some domains were lost due to small exon length. In addition, selected cases of domain loss were additionally tested by BLAST searches seeded with human orthologs. No disagreement with HMMER search of PFAM domains was found.

Ostrander et al. recently reviewed the case of the Syrian hamster sarcoma [7]. It appears that several research groups studied different cases of hamster tumors. Apparently, the SV40 virus can cause sarcomas, but it is not clear if a viral agent caused the original reticulum cell sarcoma studied in the article we cite. We added a comment that the viral origin of such tumors is an alternative possibility to our introduction.

The re-evolution of genes to regain multicellularity is an interesting question. However, it is not clear how to search for such genes. It is unlikely that such re-evolved genes share sequence similarity to those that were required for multicellularity before, if the original genes were lost, even if they provide the same functions. It is also worth mentioning that the multicellularity of Myxosporea is quite different from that of other Cnidaria.

We thank the reviewer for noticing an error. In a 2007 review Grosberg et al. write that “Multicellular organisms independently originated at least 25 times from unicellular ancestors” [49]. In a 2013 review Parfrey et al. state that “Multicellularity has arisen more than 25 times across the eukaryotic tree of life and in all of the major clades” [50]. We corrected the text.

### Reviewer 3 Dr. Gregory M Woods

#### Initial submission

The hypothesis is that “Some simplified relatives of complex metazoans can have a tumor origin.” This is intriguing, and the authors provide some supporting evidence. The authors propose that “Relatives of more complex metazoans have genomic alterations typical for cancer progression (such as deletions of universal apoptosis genes).” However, if this was the case, some control would be required to prevent continuous “cancerous” growth. The deletion of apoptotic genes appears to be the authors’ major support of the “tumor” origin theory. The manuscript proposes an original hypothesis that initially appears implausible, but the authors propose some logical steps from cancer cell to a species. But the evidence is not compelling and selective (apoptotic genes).

This is an original hypothesis, which requires an open mind to seriously consider. Major recommendations Page 10 line 11 “Thus, we can put forward the hypothetical steps of Myxosporea evolution. First, a parasitic polypodium-like organism produced a tumor in a fish host. Some genes related to apoptosis and cell-cycle control were lost. This tumor acquired transmissibility as in known mammalian and molluscan transmissible cancers. Two different evolutionary trends followed and produced the modern Myxosporea. One trend was caused by the cancerous origin of Myxosporea species: multiple genes involved in apoptosis, cell-cycle suppression and associated pathways were abruptly lost. A different trend was directed towards a de novo formation of multicellularity resulting in the currently observed strange organisms with three cells stages and bizarre cell aggregates like syncitia with whole cells inside other cells.” Why was it necessary for the tumor to be polypodium like? How does this differ from speciation or evolution? Was this tumor polypodium in origin, or did it transform a host cell? How did the tumor acquire transmissibility? If it was a “mutated” parasite, then it could be transmitted. But if it was a transformed somatic, or even stem cells as the authors intimate, then a mechanism of transfer is required. Further, if it was a somatic cell, how did it acquire genes to allow it to survive outside the host, in parasitic form? Genes controlling apoptosis are essential for the ‘sculpting’ of multicellular organisms, especially those with defined organs. How can Myxosporea survive without apoptotic genes? Clearly, Myxosporea exist, so there must be an alternative mechanisms. Evidence was provided for the loss of apoptotic related genes and p53, but what were the associated pathways. What were the mechanisms of de novo formation of multicellularity. A reference was quoted but mechanism not explained. It wasn’t clear whether the tumor cell was initially a parasite or a somatic cell. If the former, how is this different from ‘evolution’ and if the latter, how was the stem cell sculpted into a multi-cellular organism. Minor recommendations Page 2, line 18 – “Given the ability of some tumors to survive for thousands of years [5]” – One tumor, CTVT, has existed for thousands of years. Page 2 line 19 their progenitors as in the case of the canine transmissible venereal tumor [10], it is possible for them to evolve into a new species. How can this be possible? Big jump from complex mammals to simple metazoans.

#### Authors response (initial submission)

We thank Dr. Woods for his comment and understand his concerns. We would like to answer his questions in a point-by-point manner.

1. Why was it necessary for the tumor to be *Polypodium* like?

We do not state that the tumor needs to be *Polypodium* like. We merely state that *Polypodium* is the closest phylogenetic group relative of Myxozoa among presently known Сnidaria. If Myxosporea evolved as a SCANDAL, then their closest relatives give us our best guess about how their ancestors looked like.

2. How does this differ from speciation or evolution?

The difference is that there was (as we hypothesize) a catastrophic simplification of Myxosporean ancestors through a tumor stage, followed by a de novo acquisition of multicellularity. This would be a very unusual process of evolution and speciation.

3. Was this tumor *Polypodium* in origin, or did it transform a host cell?

We hypothesize that it was a tumor of *Polypodium* that spread to the host. Myxosporeans are genetically related to other Cnidaria, not their fish or annelid hosts. We clarified this in the text.

4. How did the tumor acquire transmissibility?

We do not know the exact mechanism. However, we know that some tumors acquire transmissibility through some mechanism.

5. Further, if it was a somatic cell, how did it acquire genes to allow it to survive outside the host, in parasitic form?

We believe this point is similar to point 3.

6. e6. Genes controlling apoptosis are essential for the ‘sculpting’ of multicellular organisms, especially those with defined organs. How can Myxosporea survive without apoptotic genes?

They do not have defined organs and their multicellularity is different from that of most Metazoa.

7. What were the mechanisms of de novo formation of multicellularity

We do not know.

8. It wasn’t clear whether the tumor cell was initially a parasite or a somatic cell

Our hypothesis is that a Cnidaria somatic cell became first a tumor and then a transmissible parasite. Actually, there could be two different scenarios: 1) a free-living Cnidarian acquired a transmissible tumor that later infected a different species (fish or annelid). 2) a parasitic Cnidarian acquired a transmissible tumor that later adopted transmission between host species. In our example scenario, we preferred the second possibility because the fish parasite *Polypodium* is the closest known phylogenetic relative of Myxozoa. Myxosporea and Malacosporea are fish parasites.

9. How can this be possible? Big jump from complex mammals to simple metazoans.

We know that multicellularity has evolved independently more than a few times on the tree of life. Therefore, given the possibility for a line of transmissible cancer cells to exist for thousands of years, it is possible that they can exist longer, long enough to evolve multicellularity. We do not claim that this is a probable or likely scenario, but we do believe that it is possible. If complex mammals can acquire transmissible tumors, why not Cnidaria?

We thank the reviewer for the suggested minor correction about the CTVT tumor sentence.

### Reviewer 3 (revision 1): Gregory M woods

Although the SCANDAL hypothesis is unlikely, it is intriguing, and worth publishing as a hypothesis. The authors responses were minimal but generally adequate, with two minor exceptions (below). How does this differ from speciation or evolution? “The difference is that there was (as we hypothesize) a catastrophic simplification of Myxosporean ancestors through a tumor stage, followed by a de novo acquisition of multicellularity. This would be a very unusual process of evolution and speciation.” Agreed and understood, but the authors’ hypothesis is also a “very unusual process”. There was no mechanism mentioned for acquisition of multicellularity. It would have been beneficial to respond to the question following the above paragraph in the text. If I have misinterpreted, especially as evolution is frequently mentioned, others may. How did the tumor acquire transmissibility? We do not know the exact mechanism. However, we know that some tumors acquire transmissibility through some mechanism. But please provide potential examples rather than “some mechanisms”. Transmissibility is central to the overall hypothesis.

#### Author’s response

We thank the reviewer for his further comments about our hypothesis. We think that we were not correctly understood. The phrase “very unusual process” was referring to our hypothesis. The process we describe is evolution and it is speciation, but with an unusual detail: a multicellular organism gets a tumor, the tumor evolves and becomes transmissible, and the transmissible tumor evolves and becomes a new multicellular species. Examples of first two stages exist and we mention them in our introduction (the Tasmanian devil facial tumor disease, the canine transmissible venereal tumor, transmissible cancers of bivalve mollusks). Examples of single cell organisms evolving into multicellular species are also known. Here we refer to reviews [49, 50]. However, it is yet unknown if a sequence of all three stages has occurred in nature. Our comparative genomic analysis suggests that this could be true for Myxosporea evolution. However, additional studies are required to confirm or refute this hypothesis and to propose a more detailed mechanism of multicellularity acquisition in this case (if the hypothesis is true).

## Additional file

Additional file 1: **Table S1.** A comparison of SCANDAL candidates and control species in terms of PFAM domains. We used the PFAM list from a “census of human cancer genes” [22] and performed a PFAM search for the term “apoptosis”. 409 domains were present in either of the two lists and present in 15 or more of 29 the control Metazoan species. PFAM domains in unicellular basal Holozoans are shown on the right. (XLSX 86 kb)
